# Multiple Site-Specific One-Pot Synthesis of Two Proteins by the Bio-Orthogonal Flexizyme System

**DOI:** 10.3389/fbioe.2020.00037

**Published:** 2020-02-04

**Authors:** Qiuyun Xiao, Zihan Liu, Xuan Zhao, Hai Xiong

**Affiliations:** ^1^Institute for Advanced Study, Shenzhen University, Shenzhen, China; ^2^Key Laboratory of Optoelectronic Devices and Systems of Ministry of Education and Guangdong Province, College of Optoelectronic Engineering, Shenzhen University, Shenzhen, China

**Keywords:** genetic encoding, post-translational modifications, cell-free translation, flexizyme, acetyl-lysine, histone

## Abstract

Lysine acetylation is a reversible post-translational modification (PTM) vastly employed in many biological events, including regulating gene expression and dynamic transitions in chromatin remodeling. We have developed the first one-pot bio-orthogonal flexizyme system in which both acetyl-lysine (AcK) and non-hydrolysable thioacetyl-lysine (ThioAcK) were site-specifically incorporated into human histone H3 and H4 at different lysine positions *in vitro*, either individually or in pairs. In addition, the high accuracy of this system moving toward one-pot synthesis of desired histone variants is also reported.

## Introduction

Mononucleosomes, the fundamental repeating unit of chromatin, have successfully colonized eukaryotic cells. Each mononucleosome consists of genomic DNA wrapped around an octamer of four core histones (H2A, H2B, H3, and H4) (Luger et al., 1997; Chatterjee and Muir, 2010; Liszczak et al., 2018). The core histones are decorated by several post-translational modifications (PTMs) at their *N*-terminal tails. Core histones also have global effects on dynamic modulation of chromatin structure and function. As a result, they are thought to have had a major effect in the cellular gene expression program (Kouzarides, 2007; Huang et al., 2015). Over the years numerous reports have been published on the histone acetylation sites and their contribution to diverse biological processes. It is now widely accepted that lysine acetylation has broad regulatory functions (Choudhary et al., 2009; Tan et al., 2011; Saredi et al., 2016; Chirichella et al., 2017). The genetic code expansion strategy has been utilized to co-translationally incorporate individual non-canonical amino acids (ncAAs) into histone proteins at specific lysine residue sites, which are later subject to PTM studies (Liu and Schultz, 2010; Lang and Chin, 2014; Fan et al., 2015; Chin, 2017; Wang, 2017; Liu et al., 2018; Zhang et al., 2018; Oller-Salvia and Chin, 2019; Reille-Seroussi et al., 2019). It has been reported that the crosstalk between histone lysine acetylation and other PTMs is momentous in chromatin-based control and in shaping inheritable epigenetic programs (Suganuma and Workman, 2008; Zippo et al., 2009; Liu et al., 2014). Moreover, the simultaneous incorporation of multiple distinct ncAAs of PTMs into one protein is desirable. As noted above, for simultaneous site-specific incorporation of several ncAAs into proteins, the dual genetic code expansion strategy has been applied to design two different stop codons or one stop codon together with one four-base codon (Hoesl and Budisa, 2011; Rashidian et al., 2013; Zang et al., 2015; Luo et al., 2016; Venkat et al., 2018; Wollschlaeger et al., 2018; Zheng et al., 2018).

## Materials and Methods

### Synthesis of the Compounds

AcK-DBE and ThioAcK-DBE were synthesized according to published procedures (Xiong et al., 2016).

### Construction of H3wt and H4wtand Variants

The human H3wt and H4wt gene fragments were purchased from Life Technologies and were expressed in *Escherichia coli*. The plasmids pUC-H3wt and pUC-H4wt were derived from the plasmid pUC-19 (Invitrogen). Four stop codon mutations, H3K27(UAG), H4K16(UAG), H4K91(UGA), H4K16(UAG)/K91(UGA) were introduced to the pUC-H3wt and pUC-H4wt gene with the MutantBEST kit (TaKaRa).

### U73A-tRNA^sep^ Preparation and Purification

T7 RNA polymerase run-off transcription was prepared *in vitro* as reported previously (Xiong et al., 2016). U73A-tRNA^sep^ gene together with the T7 promoter was amplified by PCR, and the fragments were cloned to vector pUC19. The U73A-tRNA^sep^ transcript was purified by electrophoresis on denaturing polyacrylamide gels and full-length tRNA extracted by 250 mM NaOAc in 75% EtOH.

### Aminoacylation Assay of U73A-tRNA^sep^

Aminoacylation of U73A-tRNA^sep^ was achieved using a previous description of the process (Xiong et al., 2016): 2 μL of 250 μM U73A-tRNA^Sep^, 2 μL of 500 mM HEPES-KOH buffer (pH 7.2), 2 μL of 250 μM dFx, and 6 μL of nuclease free H_2_O were mixed well, and heated at 95°C for 3 min, then the reaction mixture was slowly cooled at room temperature for more than 5 min. A 2 μL of 3 M MgCl_2_ was added into the above mixture, and then incubated at room temperature for 5 min followed by incubation on ice for 3 min. To start the acylation reaction, 4 μL of 25 mM of acid substrate in DMSO was added, and the mixture was incubated on ice for 6 hr. To stop the reaction, 40 μL of 0.6 M sodium acetate was added, and followed by 200 μL ethanol for precipitation. Centrifuge the samples at 14,000 rpm for 15 min at 25°C. 70% ethanol containing 0.1 M NaCl was used to rinse the pellet twice, and the pellet was dissolved in 10 μL of 10 mM sodium acetate. A 1 μL of this solution was mixed with 1 μL of acid PAGE loading buffer [contained 150 mM sodium acetate (pH 5.2)], and analyzed by 12% denaturing PAGE [contained 50 mM sodium acetate (pH 5.2)]. TBE buffer that contained 0.1 M sodium acetate was used as running buffer. After electrophoresis, the gel was washed with 50 mL of 1 × TBE by gently shaking for 10 min. Further, the gel was stained with 20 mL of ethidium bromide gel-staining solution by gently shaking for 10 min, washed briefly with 50 mL of RNase-free water and with 50 mL of 1 × TBE by gently shaking for 5 min. Finally, the gel image was scanned by using a fluorescence imaging system. To determine the yield of acylation, the bands were quantified by corresponding to free and acylated tRNA.

### Incorporations of AcK and/or ThioAcK on Specific-Site of Human Histone H3 or H4 by Using Cell-Free Translation

To carry out protein modification, a cell-free protein synthesis of non-canonical amino acids were used with the PURExpress^®^ ΔRF123 Kit (E6850, BioLabs Inc.). According to the manual, 2 μL DNA template (150 ng/μL) and 1 μL acylated tRNA were made up to 25 μL volume. Then, the mixture was incubated at 37°C for 4 hr, stopping the reaction by cooling. The products were stored at −20°C until use (Murakami et al., 2006; Goto et al., 2011; Xiong et al., 2016).

### Western Blotting Assay

Anti-acetyl histone H3K27, H4K16, and H4K91 antibodies (Abcam, ab4729, ab109463, ab4627) are special site-selective for the acetylated histone modifications, and they can be performed to distinguish some acetylated histone variants and non-acetylated histone proteins such as commercial histone H3 and commercial histone H4.

### HPLC-MS/MS of Demonstrated the Incorporation of ThioAcKand/orAcK in Histone H3 and H4

The Coomassie blue stained SDS-gel that contained histone H3 and H4 variants were digested and analyzed by LC-MS/MS analyses on Q Exactive (Thermo Fisher) and Easy-nLC 1000 (Thermo Fisher).

## Results and Discussion

**FIGURE 1:**
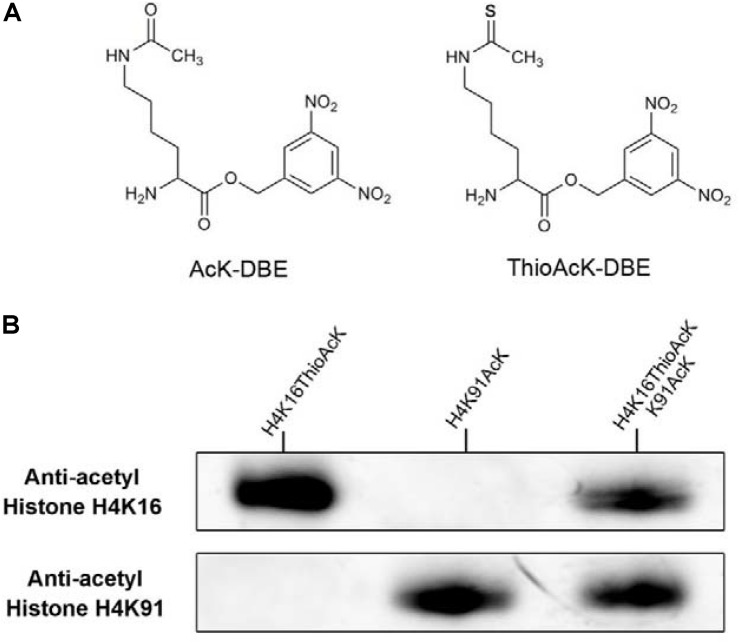
**(A)** Structure of acetyl-lysine 3,5-dinitrobenzyl ester (AcK-DBE) and thioacetyl-lysine 3,5-dinitrobenzyl ester (ThioAcK-DBE). **(B)** Western Blots of single or dual ncAAs (AcK, ThioAcK) residues into H4 variants (H4K16_ThioAck/H4K91_AcK) with site-specific anti-acetyl histone antibodies (Abcam, ab109463, ab4627). 10 μL of the corresponding PURE reaction solution was loaded into each lane.

By taking advantage of the bio-orthogonal Flexizyme system (Murakami et al., 2006; Terasaka et al., 2014), we previously developed a novel approach for the dual site-specific incorporation of acetyl-lysine (AcK) and non-hydrolysable thioacetyl-lysine (ThioAcK) at different lysine positions into the full-length histone H3 *in vitro* (Xiong et al., 2016). With our experience and as a part of our ongoing research program, we expanded this strategy to site-specific one-pot synthesis of two histones (H3 and H4) for simultaneous incorporation of AcK and ThioAcK at different lysine positions *in vitro*, either individually or in pairs. To generate full-length H3 and H4 histones containing the AcK and non-deacylatable ThioAcK, we used dinitro-flexizyme (dFx) to acetylate transfer RNA (tRNA) with the 3,5-dinitrobenzyl esters of N^ε^-acetyl-lysine (AcK-DBE) and N^ε^-thioacetyl-lysine (ThioAcK-DBE, Figure 1A). We also demonstrated their utility in accurate one-pot synthesis of two desired histones through the Western Blot assay and Tandem-mass spectrometry (MS). Further, the results reveal that the significant challenge of homogeneously generating two proteins modified with different ncAAs at multiple specific sites with high over-expression in one bio-translation system is solved through this method.

**FIGURE 2:**
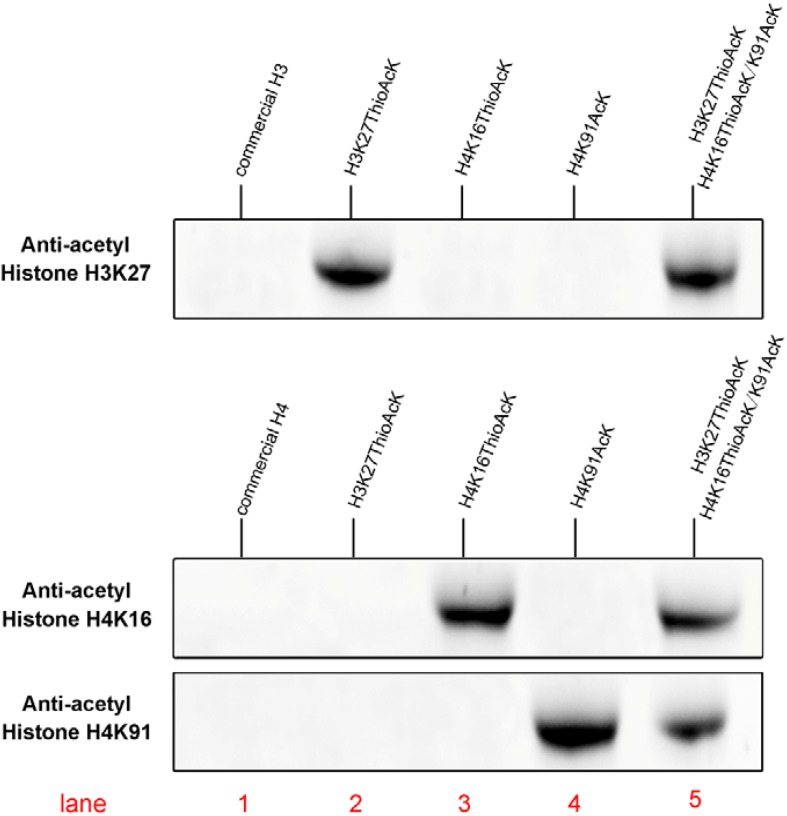
Western Blots of single or multiple ncAAs residues into H3K27_ThioAcK variant on H3 with anti-acetyl histone H3K27AcK antibody, or variants (H4K16_ThioAcK, H4K91_AcK or H4K16_ThioAcK/H4K91_AcK) on H4 with anti-acetyl histone H4K16AcK and H4K91AcK antibodies. 10 μL of the corresponding PURE reaction solution was loaded into each lane. Lane 1: commercial histone H3 or H4 (10 μg) as antibody control.

Acetylation of histone H4 at lysine 16 (H4K16AcK) is an important PTM for regulating gene activation and silencing (Shogren-Knaak et al., 2006; Williams et al., 2009; Zippo et al., 2009; Bai et al., 2018). However, H3K27ac differentiates active enhancers from poised enhancer elements that contain H3K4me1 alone (Creyghton et al., 2010). Two targets histone H4K16/K91 and H3K27 were selected since the residue sites located on the N-terminal tail are accessible to chromatin when assembled into nucleosomes. To further investigate the incorporation of ThioAcK and AcK modifications in H4 histone proteins, we directed ThioAcK or AcK to positions K16 or K91 in the human histone H4 by UAG or UGA suppression with a dinitro-Flexizyme (dFx) aminoacylated U73A-tRNA^Sep^_CUA_ or U73A-tRNA^Sep^_UCA_ anticodon in the PURExpress (NEB) *in vitro* translation system. Upon incorporation with site-specific anti-histone H4K16ac and H4K91ac antibodies (Abcam ab109463, ab4627), we observed high efficiency of incorporation of ThioAcK or AcK at K16_ThioAcK (UAG) or K91_AcK(UGA), respectively (Figures 1B, 2, lane 3 and lane 4). At the same time, we also directed ThioAcK to position K27 in the human histone H3 by UAG suppression with the dFx system. Incorporation with a site-specific anti- histone H3K27ac antibody (Abcam ab4729) detected a high efficiency of incorporation of ThioAcK at K27_ThioAcK (UAG) (Figure 2, lane 2). An independent set of experiments revealed that the incorporation of ThioAcK and AcK in histones H4 or H3 at different positions has strong affinities for AcK-specific antibodies.

After successful incorporation of ThioAcK or AcK into full-length H4 or H3, we had chosen K16/K91 in histone H4 to incorporate ThioAcK and AcK simultaneously by a combination of UAG and UGA codon suppression. The PUREPRESS system which lacks release factor 1 and 2 (PURE ΔRF1, 2) was used to synthesize the full-length H4 with two different modification sites. Due to the length limit of the DNA fragment, the protein expression could be terminated naturally. The incorporation of ThioAcK and AcK at position K16 [H4K16_ThioAcK (UAG)] and K91 [H4K91_AcK (UGA)] was confirmed by site-specific anti-histone H4K16AcK and H4K91AcK antibodies (Abcam). Experimental results showed that ThioAcK incorporation in histone H4K16 and AcK incorporation in histone H4K91 have strong affinities for AcK-specific antibodies (Supplementary Figure S1). Furthermore, we found that a high efficiency of dual genetic incorporation of ThioAcK and AcK is the same as that of a single ncAA incorporation.

Inspired by the one-pot synthesis of chemicals, we attempted to generate two full-length histones H3 and H4 in the same PURExpress system. We incorporated ThioAcK and AcK in histone H3 at position K27 [H3K27_ThioAcK (UAG)] and histone H4 at positions K16/K91 [H4K16_ThioAcK (UAG)/K91_AcK (UGA)] by UAG and UGA suppression with the PUREPRESS system lacking release factor 1 and 2. Western Blots showed observable signals in nearly all the histone H3 and H4 modifications. For the site-specific anti-acyl histone antibodies (H3K27, H4K16, and H4K91), three identical one-pot experiments were performed on the above three antibodies, respectively. Lane 5 showed the same efficiency of ThioAcK and AcK incorporation by one-pot synthesis compared to lanes 2–4 (Figure 2).

**FIGURE 3:**
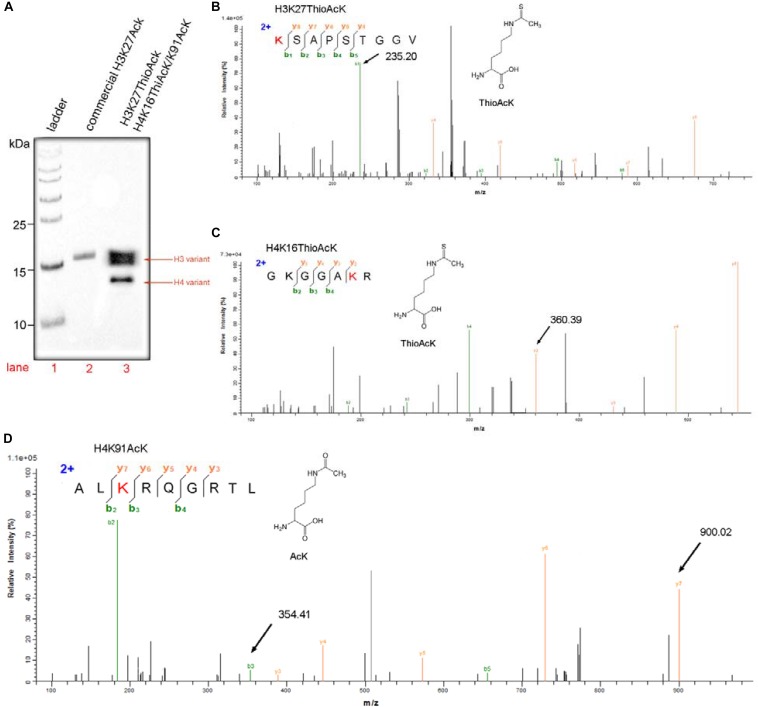
One-pot incorporation multiple ThioAcK and AcK residues into histone H3 and H4 variants (H3K27_ThioAcK and H4K16_ThioAcK/K91_AcK). **(A)** Western Blots of multiple PTMs on H3 and H4 with the site-specifically anti-ackyl histone antibodies (H3K27, H4K16 and H4K91). 10 μL of the corresponding PURE reaction solution was loaded. Lane 1: commercial protein marker as ladder. **(B)** MS/MS spectra of tryptic peptideK (ThioAcK) SAPSTGGV digestion for H3K27_ThioAcK at the site of K27. **(C)** MS/MS spectra of tryptic peptides GKGGAK (ThioAcK) R digestion at K16 of the K16 ThioAcK and **(D)** ALK(AcK)RQGRTL digestion at K91 of the K91AcK.

Western Blot was also used widely to evaluate the semi-quantitative concentrations of specific proteins. Three above histone antibodies, in turn, were performed on the one-pot system, and a commercial protein marker was used as a ladder (Figure 3A). The protocol is listed in detail in the supporting information. Western Blots were carried out on 15% SDS-PAGE gels loaded with 5 μg of a commercial H3K27AcK as a standard (Lane 2, Figure 3A) and 10 μL of total histone H3 and H4 modifications products (Lane 3, Figure 3A). The quantity of target H3 and H4 variants (H3K27_ThioAcK, H4K16_ThioAcK/H4K91_AcK) prepared by one-pot synthesis was calculated and analyzed using ImageJ2 software. The relative band intensity of H3 and H4 variants were 4.60 and 1.69 folds of the commercial H3K27AcK, respectively. Based on the loading of 5 μg commercial H3K27AcK, the yields for our experiment is about 2.298 mg/mL for the H3 variant and 0.845 mg/mL for the H4 variant. The mechanistic experiments showed that AcK incorporation in H3 at the position K27 has a stronger affinity for AcK-specific antibodies than the ThioAcK and AcK incorporation in H4 at the positions K16/K91 (Figure 3A).

As shown in Figure 3, a variety of multiple histone variants (H3K27_ThioAcK, H4K16_ThioAcK/H4K91_AcK) was prepared efficiently by one-pot synthesis. MS/MS analysis also showed the correct mass spectra for the three digested peptides (Figures 3B–D). MS/MS fragmentation of three peptides from enzymolysis assigned the K (ThioAcK) SAPSTGGV digestion for H3K27_ThioAcK at the position of K27 [Mobs = 235.2032 Da; Mcalc = 235.20 Da], and GKGGAK (ThioAcK) R digestion at the position of K16 [Mobs = 360.3894 Da; Mcalc = 360.39 Da] as well as ALK (AcK) RQGRTL at the position of K91 [Mobs = 900.0234 Da; Mcalc = 900.02 Da] for H4K16_ThioAcK/H4K91_AcK (signal arrow in Figure 3, and blue color mark in Supplementary Tables S1–S3).

To the best of our knowledge, this is the first report on lysine modification in full-length human histone H4 with combinations of ThioAcK and AcK. In the best-case scenario, the present modern approach provides an efficient method to generate two full-length histones H3 and H4 simultaneously by incorporating similar chemical structures of ThioAcK and AcK using one-pot bio-orthogonal flexizyme system. We anticipate that investigations in these areas can be used efficiently to synthesize PTM of other histones or non-histone proteins, which might allow further study of their crosstalk and aid key epigenetic research in chromatin remodeling and nucleosome recombination.

## Data Availability Statement

All datasets generated and analyzed for this study are included in the article/Supplementary Material.

## Author Contributions

HX conceived the experiments. QX conducted all the experiments. ZL and XZ helped to discuss the results. HX and QX drafted the manuscript.

## Conflict of Interest

The authors declare that the research was conducted in the absence of any commercial or financial relationships that could be construed as a potential conflict of interest.
