# Supplementary material for: Multiple Site-Specific One-Pot Synthesis of Two Proteins by the Bio-Orthogonal Flexizyme System
Source: Front Bioeng Biotechnol. 2020 Feb 4;8:37. doi: 10.3389/fbioe.2020.00037 (PMC7010957; doi:10.3389/fbioe.2020.00037)
Supplement: Supplementary file 1 [file Data_Sheet_1.doc]

Supplementary Material

# Supplementary Data

**Human H3wt sequence：**

1 ATGGCACGTACCAAACAGACCGCACGTAAAAGCACCGGTGGTAAAGCACCGCGTAAACAG

1 M A R T K Q T A R K S T G G K A P R K Q

61 CTGGCAACCAAAGCAGCCCGTAAAAGCGCACCGAGTACCGGTGGTGTTAAAAAACCGCAT

21 L A T K A A R K S A P S T G G V K K P H

121 CGTTATCGTCCGGGTACAGTTGCACTGCGTGAAATTCGTCGTTATCAGAAAAGTACCGAA

41 R Y R P G T V A L R E I R R Y Q K S T E

181 CTGCTGATTCGTAAACTGCCGTTTCAGCGTCTGGTTCGTGAAATTGCACAGGATTTCAAA

61 L L I R K L P F Q R L V R E I A Q D F K

241 ACCGATCTGCGTTTTCAGAGCGCAGCAATTGGTGCACTGCAAGAAGCAAGCGAAGCATAT

81 T D L R F Q S A A I G A L Q E A S E A Y

301 CTGGTTGGCCTGTTTGAAGATACCAATCTGTGTGCAATTCATGCCAAACGTGTTACCATT

101 L V G L F E D T N L C A I H A K R V T I

361 ATGCCGAAAGATATTCAGCTGGCACGTCGTATTCGTGGTGAACGTGCATAA

121 M P K D I Q L A R R I R G E R A *

**Human H4wt DNA sequence：**

1 ATGTCCGGCAGAGGAAAGGGCGGAAAAGGCTTAGGCAAAGGGGGCGCTAAGCGCCACCGC

1. M S G R G K G G K G L G K G G A K R H R

61 AAGGTCTTGAGAGACAACATTCAGGGCATCACCAAGCCTGCCATTCGGCGTCTAGCTCGG

21 K V L R D N I Q G I T K P A I R R L A R

121 CGTGGCGGCGTTAAGCGGATCTCTGGCCTCATTTACGAGGAGACCCGCGGTGTGCTGAAG

41 R G G V K R I S G L I Y E E T R G V L K

181 GTGTTCCTGGAGAATGTGATTCGGGACGCAGTCACCTACACCGAGCACGCCAAGCGCAAG

61 V F L E N V I R D A V T Y T E H A K R K

241 ACCGTCACAGCCATGGATGTGGTGTACGCGCTCAAGCGCCAGGGGCGCACCCTGTACGGC

81 T V T A M D V V Y A L K R Q G R T L Y G

301 TTCGGAGGCTAG

101 F G G *

**U73A-tRNA^sep^**

5’-GCCGGGGTAGTCTAGGGGTTAGGCAGCGGACTCTAGATCCGCCTTACG

TGGGTTCAAATCCCACCCCCGGCACCA-3’

**5****’-UTR sequence of Forward Primer:**

5’-GCGAATTAATACGACTCACTATAGGGCTTAAGTATAAGGAGGAAAAAATATGAGTA

AAGGAGAAGAACTTTTCACTGGA

**3’-UTR sequence of Reverse Primer:**

5’-AAACCCCTCCGTTTAGAGAGGGGTTATGCTAGTTAGGATCCTTTGTAGAGCTCATCCA

TGCC

# Supplementary Tables

**Supplementary Table S1**. Assigned MS/MS fragments resulting from fragmentation of the tryptic peptide K(ThioAcK)SAPSTGGV of the histone H3K27_ThioAcK digestion; Precursor ion: M_obs_ = 861.2300 Da (red color mark); M_calc_ = 860.88 Da.

| # | b | b^++^ | b^+^ | b^*++^ | b^0^ | b^0++^ | seq. | y | y^++^ | y^+^ | y^*++^ | y^0^ | y^0++^ | # |
| --- | --- | --- | --- | --- | --- | --- | --- | --- | --- | --- | --- | --- | --- | --- |
| **1** | **235.2032** | 118.1067 |  |  |  |  | **K** | **861.2300** | 431.1201 | 844.2034 | 422.6053 | 843.2194 | 422.1135 | **9** |
| **2** | 322.3343 | 161.6723 | 305.3077 | 153.1575 |  |  | **S** | 674.9574 | 337.9838 | 657.9308 | 329.4690 | 656.9468 | 328.9772 | **8** |
| **3** | 393.8803 | 197.4453 | 376.8537 | 188.9305 |  |  | **A** | 587.8066 | 294.4084 | 570.7800 | 285.8936 | 569.7960 | 285.4018 | **7** |
| **4** | 493.5326 | 247.2714 | 476.5060 | 238.7566 | 475.5220 | 238.2648 | **P** | 516.7539 | 258.8821 | 499.7273 | 250.3673 | 498.7433 | 249.8755 | **6** |
| **5** | 580.3109 | 290.6601 | 563.2843 | 282.1458 | 562.3003 | 281.6540 | **S** | 419.6725 | 210.3414 | 402.6459 | 201.8266 |  |  | **5** |
| **6** | 681.2609 | 341.1356 | 664.2343 | 332.6208 | 663.2503 | 332.1290 | **T** | 332.5030 | 166.7566 | 315.4764 | 158.2418 |  |  | **4** |
| **7** | 680.5511 | 340.7807 | 663.5245 | 332.2659 | 662.5405 | 331.7741 | **G** | 231.4985 | 116.2544 | 214.4719 | 107.7396 |  |  | **3** |
| **8** | 795.3294 | 398.1698 |  |  |  |  | **G** | 174.3294 | 87.6698 | 157.3028 | 79.1550 |  |  | **2** |
| **9** |  |  |  |  |  |  | **V** | 117.1289 | 59.06955 |  |  |  |  | **1** |

**H4K16ThioAcK(UAG)**

**Supplementary Table S2**. Assigned MS/MS fragments resulting from fragmentation of the tryptic peptide GKGGAK(ThioAcK)R of the histone H4K16_ThioAcK digestion; Precursor ion: M_obs_ = 730.8097 Da (red color mark); M_calc_ = 730.79 Da.

| # | b | b^++^ | b^+^ | b^*++^ | b^0^ | b^0++^ | seq. | y | y^++^ | y^+^ | y^*++^ | y^0^ | y^0++^ | # |
| --- | --- | --- | --- | --- | --- | --- | --- | --- | --- | --- | --- | --- | --- | --- |
| **1** | 57.1650 | 29.0862 |  |  |  |  | **G** | **730.8097** | 365.9086 | 713.7831 | 357.3953 | 712.7991 | 356.9033 | **7** |
| **2** | 185.3419 | 93.1747 | 168.3153 | 84.1577 |  |  | **K** | 673.7227 | 337.3651 | 656.6961 | 328.8518 | 655.7121 | 328.3598 | **6** |
| **3** | 242.3939 | 121.7007 | 225.3673 | 112.6837 |  |  | **G** | 545.5945 | 273.3010 | 528.5679 | 264.7877 | 527.5839 | 264.2957 | **5** |
| **4** | 299.4356 | 150.2215 | 282.4090 | 141.2045 | 281.4250 | 140.7125 | **G** | 488.5278 | 244.7676 | 471.5012 | 236.2543 |  |  | **4** |
| **5** | 370.5226 | 185.7650 | 353.4960 | 176.7480 | 352.5120 | 176.2560 | **A** | 431.4709 | 216.2392 | 414.4443 | 207.7259 |  |  | **3** |
| **6** | 556.6926 | 278.8500 | 539.6660 | 269.8330 | 538.6820 | 269.3410 | **K** | **360.3894** | 180.6984 | 343.3628 | 172.1851 |  |  | **2** |
| **7** | 712.8806 | 356.9440 | 695.8540 | 347.9270 | 694.8700 | 347.4350 | **R** | 174.2125 | 87.6100 | 157.1859 | 79.0967 |  |  | **1** |

**H4K91AcK(UGA)**

Supplementary Table S3. Assigned MS/MS fragments resulting from fragmentation of the tryptic peptide ALK (AcK) RQGRTL of the histone H4K91_AcK digestion; Precursor ion: M_obs_ = 900.0234 Da (red color mark); M_calc_ = 900.02 Da

| # | b | b^++^ | b^+^ | b^*++^ | b^0^ | b^0++^ | seq. | y | y^++^ | y^+^ | y^*++^ | y^0^ | y^0++^ | # |
| --- | --- | --- | --- | --- | --- | --- | --- | --- | --- | --- | --- | --- | --- | --- |
| **1** | 71.1040 | 36.0557 | 54.0774 | 27.5424 |  |  | **A** | 1084.2461 | 542.6268 | 1067.2195 | 534.1135 | 1066.2355 | 533.6215 | **9** |
| **2** | 184.2452 | 92.6263 | 167.2186 | 84.1130 | 166.2346 | 83.6210 | **L** | 1013.1646 | 507.0860 | 996.1380 | 498.5727 | 995.1540 | 498.0807 | **8** |
| **3** | 354.4102 | 177.7088 | 337.3836 | 169.1955 | 336.3996 | 168.7035 | **K** | 900.0234 | 450.5154 | 882.9968 | 442.0021 | 882.0128 | 441.5101 | **7** |
| **4** | 510.5860 | 255.7967 | 493.5594 | 247.2834 | 492.5754 | 246.7914 | **R** | 729.8509 | 365.4292 | 712.8243 | 356.9159 |  |  | **6** |
| **5** | 638.7142 | 319.8608 | 621.6876 | 311.3475 | 620.7036 | 310.8555 | **Q** | 573.6927 | 287.3501 | 556.6661 | 278.8368 |  |  | **5** |
| **6** | 695.7559 | 348.3817 | 678.7293 | 339.8684 | 677.7453 | 339.3764 | **G** | 445.5636 | 223.2855 | 428.5370 | 214.7722 |  |  | **4** |
| **7** | 851.9684 | 426.4879 | 834.9418 | 417.9746 | 833.9578 | 417.4826 | **R** | 388.4849 | 194.7462 | 371.4583 | 186.2329 |  |  | **3** |
| **8** | 953.0696 | 477.0385 | 936.0430 | 468.5252 | 935.0590 | 468.0332 | **T** | 232.3032 | 116.6553 | 215.2766 | 108.1420 |  |  | **2** |
| **9** | 1066.2278 | 533.6176 | 1049.2012 | 525.1043 | 1048.2172 | 524.6123 | **L** | 131.1743 | 66.09085 | 114.1477 | 57.5776 |  |  | **1** |
